# New Medical Journals

**Published:** 1851-03

**Authors:** 


### ARTICLE VI.

### NEW MEDICAL JOURNALS.

*The Philadelphia Lancet.*

We are in the receipt of this new candidate for public favor which is published semi-monthly in the city of Philadelphia at one dollar per annum, and is edited by Thomas Dunn English M. D.

It is in the form of a newspaper and contains much practical information, being filled with clinical lectures, original communications, and editorial articles. It appears to be conducted with spirit and ability, and we hope it will have a long and prosperous career.

---

*The Stethoscope.*

We observe by our exchanges that a new Medical Journal called "The Stethoscope" has been issued by Dr. Gooch, Editor, in Richmond, Virginia, but not having seen a copy we are unable to speak of its character or appearance.

It would seem to us that the Great State of Virginia ought to have and support at least one good medical periodical.

